# Prevalence of Catheter-Associated Urinary Tract Infections in Neurosurgical Intensive Care Patients – The Overdiagnosis of Urinary Tract Infections

**DOI:** 10.7759/cureus.5494

**Published:** 2019-08-26

**Authors:** Stacey Podkovik, Harjyot Toor, Maya Gattupalli, Samir Kashyap, James Brazdzionis, Tye Patchana, Sruthi Bonda, Serena Wong, Christine Kang, Kevin Mo, Margaret Rose Wacker, Dan E Miulli, Sharon Wang

**Affiliations:** 1 Neurosurgery, Riverside University Health System Medical Center, Moreno Valley, USA; 2 Neurosurgery, Touro University College of Osteopathic Medicine California, Vallejo, USA; 3 Neurosurgery, St. George's University School of Medicine, St. George's, GRD; 4 Neurosurgery, Western University of Health Sciences, Pomona, USA; 5 Neurosurgery, Arrowhead Regional Medical Center, Colton, USA; 6 Infectious Disease, Arrowhead Regional Medical Center, Colton, USA

**Keywords:** urinary catheters, urinalysis, neurosurgery, urinary tract infections, intensive care units, fever, catheters, indwelling, temperature

## Abstract

Background: Hospital-acquired infections (HAIs) are profound causes of prolonged hospital stay and worse patient outcomes. HAIs pose serious risks, particularly in neurosurgical patients in the intensive care unit, as these patients are seldom able to express symptoms of infection, with only elevated temperatures as the initial symptom. Data from Center for Disease Control (CDC) and the Infectious Disease Society of America (IDSA) have shown that of all HAIs, urinary tract infections (UTIs) have been grossly over-reported, resulting in excessive and unnecessary antibiotic usage.

Methods: We conducted a retrospective analysis of 686 adult patients that were evaluated by the neurosurgery service at Arrowhead Regional Medical Center between July 2018 and March 2019. Inclusion criteria were adults greater than 18 years of age with neurosurgical pathology requiring a minimum of one full day admission to the intensive care unit (ICU), and an indwelling urinary catheter. Exclusion criteria were patients under the age of 18, those who did not spend any time in the ICU, or with renal pathologies such as renal failure.

Results: We reviewed 686 patients from the neurosurgical census. In total, 146 adult patients with indwelling urinary catheters were selected into the statistical analysis. Most individuals spent an average of 8.91 ± 9.70 days in the ICU and had an indwelling catheter for approximately 8.14 ± 7.95 days. Forty-two out of the 146 individuals were found to have a temperature of 100.4°F or higher. Majority of the patients with an elevated temperature had an infectious source other than urine, such as sputum (22 out of 42, 52.38%), blood (three out of 42, 7.14%) or CSF (one out of 42, 2.38%). We were able to find only two individuals (4.76%) with a positive urine culture and no evidence of other positive cultures or deep vein thrombosis.

Conclusions: Our analysis shows evidence to support the newest IDSA guidelines that patients with elevated temperatures should have a clinical workup of all alternative etiologies prior to testing for a urinary source unless the clinical suspicion is high. This will help reduce the rate of unnecessary urine cultures, the over-diagnosis of asymptomatic bacteriuria, and the overuse of antibiotics. Based on our current findings, all potential sources of fever should be ruled out prior to obtaining urinalysis, and catheters should be removed as soon as they are not needed. Urinalysis with reflex to urine culture should be reserved for those cases where there remains a high index of clinical suspicion for a urinary source.

## Introduction

Catheter-associated urinary tract infections (CAUTI) continue to be among the most common healthcare-associated infections in the United States. In 2011, there were an estimated two cases of CAUTIs per 1000 hospital indwelling catheter days in US acute care hospitals [1]. CAUTIs can lead to more serious complications such as sepsis and endocarditis; it is estimated that over 13,000 deaths each year are associated with healthcare-associated urinary tract infections (UTIs) [2]. Per the Infectious Disease Society of America (IDSA), a CAUTI is defined by the following criteria:1) indwelling urinary catheter for more than two days after insertion. 2) one sign or symptom including fever, suprapubic tenderness, costovertebral angle tenderness, urinary frequency or urgency or dysuria and 3) urine culture with more than 105 colony forming units (CFU)/mL of one bacterial species [2,3]. Patients with symptomatic UTIs generally present with fever, chills, urinary urgency, suprapubic tenderness, costovertebral angle tenderness, flank pain, altered mental status (in those older than 65 years of age), hypotension, and potentially, evidence of systemic inflammatory response syndrome (SIRS) [2].

These infections and their resultant fevers can be especially detrimental in patients with severe head and spine injury, who already face high rates of morbidity and mortality. Diringer et al. evaluated nearly 4300 patients in neurologic intensive care units (ICUs) and found that elevated body temperatures were independently associated with longer ICU and hospital stays, higher mortality rates, and worse outcomes. This was second only to complications from patients’ primary reason for admission [4]. Many neurosurgical patients suffer from systemic dysfunction due to their neurological injury that makes them susceptible to infections. Many cannot provide an adequate history to guide clinical decision making. Most patients require indwelling urinary catheters for a prolonged time, fostering bacterial colonization. Some may be on prophylactic antibiotics for other reasons such as standard perioperative antibiotics, drain prophylaxis, or other concurrent infection sources such as pneumonia. Such antimicrobial drug therapy can be protective for a short duration but can lead to the growth of multi-drug resistant organisms (MDRO) including Pseudomonas species, yeast, and resistant gram-negative bacilli. In addition, antibiotic use can lead to Clostridium difficile (C. diff) infections. Our goal was to determine if the cases that were being marked as CAUTIs at our hospital were due to true UTIs, or whether they were due to non-indicated urine cultures. This would allow for more accurate tailoring of infectious workup instead of culturing all possible sources, leading to unnecessary antibiotic use.

## Materials and methods

Study design and population

We conducted a retrospective analysis of 686 adult patients evaluated by the neurosurgery service at Arrowhead Regional Medical Center (ARMC) (an academic, 450-bed, Level 2 trauma center) between July 2018 and March 2019, with approval of the Institutional Review Board (IRB). Definitions for determining UTI were based on the IDSA guidelines. These guidelines state that for a patient to be diagnosed with CAUTI, they must have had a catheter placed at minimum 48 hours prior to the fever and have no alternative source of infection [3].

Inclusion criteria for the study were adults greater than 18 years of age with a neurosurgical pathology requiring a minimum of one full day admission to the ARMC ICU, and an indwelling urinary catheter for a minimum of two days. Exclusion criteria for the study were patients under the age of 18, those who did not spend any time in the ICU, or with renal pathologies such as renal failure. Certain renal pathologies can increase infectious risk or predispose to colonization, leading to abnormal results when assessing infection due to indwelling catheters.

We only screened ICU patients because that is where the majority of neurosurgical patients that have altered mentation preside. The initial search was for patients with an indwelling urinary catheter, and then the eligible patients were evaluated for their maximum temperatures (Tmax) during their stay. We recorded the results of any cultures, including blood, urine, sputum, and/or cerebrospinal fluid (CSF). The patient’s urinalysis (UA) and any evidence of pyuria were also obtained. The presenting pathologies were recorded for stratification purposes. Urinary catheter infection was determined based upon a positive urine culture, positive urinalysis, and no other coinciding positive cultures.

Statistical analysis

The data were gathered in the form of an excel spreadsheet with all protected health information (PHI) removed prior to any statistical analysis. A separate data key was kept on a separate secure internal Arrowhead server in order to be able to match the data points to particular patients if further analysis or research was indicated. Data analysis was accomplished through IBM SPSS Statistics, Version 23.0. Pearson correlation matrices were done to evaluate for any relationships within the data. A Kaplan-Meier curve estimate was created to assess the chance of neurosurgical ICU patients contracting a positive urine culture. A p-value of ≤0.05 was used for determining statistical significance.

## Results

**Figure 1:**
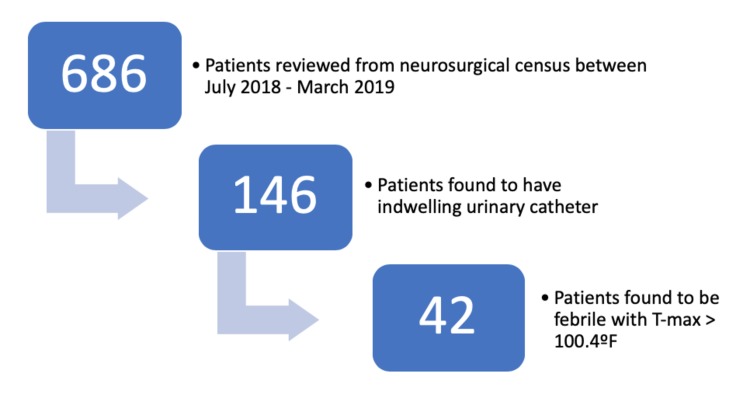
Pictorial representation of the patient selection process

We reviewed 686 patients from the neurosurgical census. In total, 146 adult patients with indwelling urinary catheters were selected for statistical analyses, contributing to 1301 hospital ICU and 1189 catheter days (Figure 1). There were 87 (59.6%) males and 59 (40.4%) females, with an overall average age of 55.48 ± 21.65 years of age (Table 1). There appeared to be a bimodal distribution in patients’ ages, with peaks at approximately 25 and 65 years of age.

**Table 1:** Demographics ICU: intensive care unit; IPH: intraparenchymal hemorrhage; SDH: subdural hematomas

Characteristic	All Patients (n=146)	Males (n=87)	Females (n=59)	p-value
Age (years)				
Mean	55.48 ± 21.65	53.64 ± 22.18	58.19 ± 20.73	0.215
Days in ICU				0.955
Mean	8.91 ± 9.70	8.87 ± 9.44	8.97 ± 10.14	
Median	5	7	5	
Days of Indwelling Urinary Catheter				0.824
Mean	8.14 ± 7.95	8.02 ± 7.64	8.32 ± 8.44	
Median	5	5	5	
T-Max (ºF)				0.05
Mean	99.69 ± 1.42	99.87 ± 1.51	99.42 ± 1.26	
Number of patients with T-Max ≥ 100ºF	42 (28.8%)	30 (34.5%)	12 (20.3%)	
Presenting Pathology				
IPH	27 (18.5%)	12 (13.8%)	15 (25.4%)	
SDH	24 (16.4%)	17 (19.5%)	7 (11.9%)	
Spine Fracture	18 (12.3%)	8 (9.2%)	10 (16.9%)	
Intracranial Tumor	18 (12.3%)	10 (11.5%)	8 (13.6%)	
Head Trauma	15 (10.3%)	10 (11.5%)	5 (8.5%)	
Other	44 (30.1%)	30 (34.5%)	14 (23.7%)	

Most individuals spent an average of 8.91 ± 9.70 days in the ICU and had an indwelling catheter for approximately 8.14 ± 7.95 days, with a median of five days (Table 1). The most prevalent admitting diagnoses were intraparenchymal hemorrhage (27 patients, 18.5%), subdural hematomas (24 patients, 16.4%), and intracranial tumors (18 patients, 12.3%) (Table 1). The overall breakdown regarding the types of positive cultures within the population is demonstrated in Table 2, with sputum as being the most common source.

**Table 2:** Frequencies of all positive cultures within the entire sample population

Characteristic	All Patients (n=146)	Males (n=87)	Females (n=59)
Number of Positive Cultures			
Urine	34 (23.3%)	14 (16.1%)	20 (33.9%)
Sputum	44 (30.1%)	26 (29.9%)	18 (30.5%)
Blood	10 (6.9%)	6 (6.9%)	4 (6.8%)
CSF	3 (2.1%)	1 (1.2%)	2 (3.4%)
Wound	1 (0.7%)	--	1 (1.7%)

Forty -two out of 146 individuals were found to have a temperature of 100.4°F or greater (mean 99.69 ± 1.421ºF) (Figure 1). The majority of the patients with an elevated temperature had an infectious source other than urine, such as sputum (22 out of 42, 52.38%), blood (three out of 42, 7.14%) or CSF (one out of 42, 2.38%) (Table 3).

**Table 3:** Frequencies of all positive cultures within only the febrile patients

Characteristic	All Patients (n=42)	Males (n=30)	Females (n=12)
Number of Positive Cultures in Febrile Patients			
Urine	12 (28.6%)	6 (20.0%)	6 (50.0%)
Sputum	22 (52.4%)	15 (50.0%)	7 (58.3%)
Blood	3 (7.1%)	1 (3.3%)	2 (16.7%)
CSF	1 (2.4%)	--	1 (8.3%)
Wound	--	--	--

**Figure 2:**
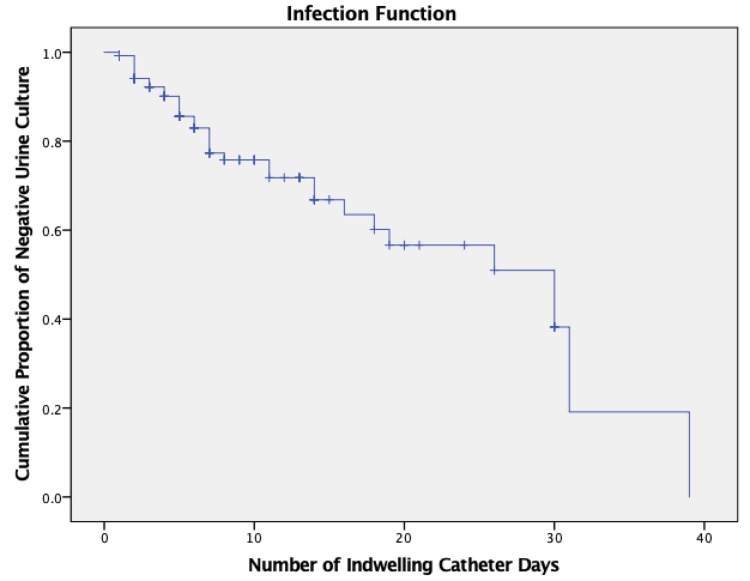
Kaplan-Meier estimate depicting the cumulative proportion of negative urine cultures over the course of indwelling urinary catheter days.

Of these, we were able to find only two individuals (4.76%) with a positive urine culture, and possible UTI, with no evidence of other positive cultures or deep vein thrombosis (DVTs). However, these two positive urine cultures were not true CAUTIs, as explained below. There was a moderate correlation (r=.399, p-value<0.001) between the number of days a urinary catheter was in place, and the maximum temperature recorded for a patient. The Kaplan-Meier estimate indicates the chance that an individual, of the total population of 146, will be free from a positive urine culture at a certain number of indwelling catheter days; with a 30-day infection-free rate of approximately 20% (Figure 2).

## Discussion

Hospital-acquired infections, especially CAUTIs, are a significant morbidity and mortality risk to the general inpatient hospital population. This risk is increased by almost five to ten-fold in those admitted to the ICU [5,6]. Risk factors found to be associated with CAUTIs are younger adults (ages 0-17) and females when accounting for variations in their underlying disease process [7]. Abulhasan et al. conducted a six-year prospective analysis of neurologic and neurosurgical ICU patients and found that they had documented CAUTIs at a rate of 3 to 5.3 infections per 1000 urinary catheter-days [8]. Klevens et al. estimated that approximately 13,000 deaths could be attributed to catheter-associated UTIs yearly [2,7,9]. Every episode of a CAUTI has been estimated to cost nearly $600 to diagnose and treat, contributing to nearly 131 million dollars in annual nationwide costs [10].

Studies such as Puri et al. and Patel et al. demonstrated that the prevalence of CAUTIs in neurosurgical and neurology patients is around 8-10%, with a mean of 8.5 to 12.5 infections per 1000 catheter days [11-13]. O’Shea et al. analyzed the prevalence of different infections in neurosurgical patients with prophylactic antibiotics in 2004 at the University Hospital of West Indies. Out of 73 patients, seven presented with urinary tract infections (about 9.5%), which is very similar to the Puri et al. study [11,14]. In a prospective study analyzing the CAUTIs in patients with indwelling catheters (> 48 hours) for 18 months, 68 out of 800 patients (8.5%) acquired a UTI. The most common organisms were: Escherichia coli (32.9%), Pseudomonas sp. (15.1%), Staphylococcus aureus (12.3%), and Candida albicans (13.7%). All gram-positive organisms were sensitive to vancomycin, while gram-negative organisms were sensitive to amikacin (sensitivity of 42%) [11]. The majority of microorganisms that cause CAUTIs are from the gastrointestinal tract; however, approximately 15% of these infections occur due to patient-to-patient transmission [10].

In our study, we evaluated 146 patients that had urine cultures obtained in the presence of an indwelling urinary catheter. We attribute our bimodal age distribution to the fact that we are a trauma center and commonly receive young patients involved in accidents or elderly people after a mechanical fall. We found two out of 42 febrile patients that had a positive urine culture, which may have attributed to a UTI; however, these individuals did not meet CAUTI criteria. We only recorded the first episode of a hospital-acquired infection, and all cultures were sent within 24 hours of the documented infection. One of the patients was an 18-year-old male after a trauma who had a fever on postoperative day (POD) 1, indicating that he did not have a urinary catheter in place for greater than 48 hours. The second patient was a 76-year-old female who presented as a Glasgow Coma Scale (GCS) 4T after an intraparenchymal hemorrhage; the family elected to pursue comfort measures. Urinalysis and urine culture were ordered upon admission for altered mental status. Therefore, this was not a CAUTI either. These findings are significantly better as compared to the national averages.

The correlation (r=0.343, p-value<0.001) between the number of days an indwelling catheter was in place, and patient temperatures is an expected finding. This number of catheter days had a higher statistically significant correlation to patient temperatures than the number of days in the ICU. This is similar to findings by Al-Hazmi et al. that demonstrated the percentage of patients that had a CAUTI between three and eight days of catheterization increased from 15% to 68% [15].

Urine cultures were considered a nominal variable, indicated by either presence or absence of a positive culture, which necessitates the use of a special (Eta) correlation statistic. There was a weak Eta correlation of 0.021 between urine culture positivity and patient temperatures. A weak correlation is expected because the vast majority of urine cultures that were found within the patient population were determined to be either asymptomatic bacteriuria (ASB). The IDSA 2019 guidelines for asymptomatic bacteriuria indicate that patients with short-term indwelling catheters have nearly a 3 to 5% cumulative daily risk of developing ASB, whereas patients with long-term catheter use have nearly a 100% chance [3,16,17]. Stickler et al. also demonstrated that 10 to 50% of patients with an indwelling urinary catheter in place for seven days would likely develop bacteriuria due to biofilm formation [18,19]. The Kaplan-Meier curve depicted in Figure 2 demonstrates that, based on the ARMC data, 80% of patients with an indwelling catheter had bacteria in their urine at 30 catheter days, increasing to 100% by 40 days, regardless of whether they were febrile or had any alternative positive cultures.

The most recent standardized infection ratio (SIR) from 2017, determined by the National Healthcare Safety Network (NHSN) was 0.850 [20]. These standardized infection ratios evaluate the number of confirmed infections relative to the number of predicted infections. The SIR at our institution was 0.734 in 2018 and 0.819 in 2017, both of which are better than the national average [21]. Significant efforts have been put in place to help reduce the number of CAUTIs within our hospital such as the Houdini Protocol, which is a nursing driven catheter removal protocol which allows nurses to remove indwelling urinary catheters if certain criteria are met. Other efforts include resident education, staff education regarding appropriate use of urinalysis and urine culture, and nurse training on proper sterile insertion techniques and maintenance.

In addition to the 146 patients included in this study, we examined the charts of the seven neurosurgical patients that were flagged as CAUTIs at our institution in 2018 to delineate a common risk factor. Of the seven patients that were noted to have CAUTIs by Medicare, four patients had concurrent pneumonia, one had multiple negative urine cultures before the final positive culture, and two were true CAUTIs. The patients all had their urinary catheters in for a minimum of five days before their positive culture result. They were all febrile above 101.0ºF with associated leukocytosis. All patients with pneumonia had exhibited signs of consolidation on chest x-ray or an increasing oxygen requirement. The argument could be made that, given this clinical picture, a urine culture was not indicated and was aberrantly ordered. The patient who had multiple negative urine cultures prior to the positive one was also an avoidable CAUTI. Once the patient had their initial fever with no suspected infectious source, and the initial urine culture had returned negative, there was no indication for nearly daily repeat urine cultures. The majority of these seven patients did not present as CAUTIs upon our analysis because they either had alternative positive cultures or were originally flagged as CAUTIs prior to the beginning timeframe of our data collection.

Neurosurgical patients are an inherently difficult population in which to diagnose UTIs due to their altered mentation. The most common reasons for urinalysis in these patients are fevers and leukocytosis. As stated earlier, there is a cumulative daily risk of developing ASB, which could lead to an errant classification of CAUTI. The diagnosis and unnecessary treatment of ASB leads to increased antibiotic resistance, unintended antibiotic side effects, exposure to more harmful infections, such as C. diff, and increased hospital costs. IDSA 2019 guidelines demonstrate strong recommendations against screening and treating ASB in cognitively impaired individuals, and to evaluate for all other possible sources prior to evaluating for a urinary source [3]. The difference between IDSA and Centers for Medicare and Medicaid Services (CMS) assessment for CAUTIs is that CMS does not factor in whether there are alternative concurrent infections besides UTI that are more likely to be the causative pathology for elevated temperatures. Our data support a stricter indication for obtaining urinalysis and urine culture in neurosurgical patients that become febrile. We recommend a diagnosis of UTI in a febrile patient with an indwelling catheter for at least five days when other diagnoses such as pneumonia, line infection, venous thrombi, or medication reactions have been ruled out through the appropriate tests.

Limitations

There were several limitations within this study. Firstly, not all of the patients had appropriate documentation. ARMC currently has a system that is transitioning from paper to electronic charting, which can contribute to loss of information. There is a certain level of human error inherently embedded in progress notes, making it difficult to analyze the clinical reasoning behind treatment plans for any given patient.

Secondly, patient outcomes were not recorded within this study despite having been shown to significantly impact elevated temperatures in the neurosurgical ICU. It may be prudent to conduct a prospective study monitoring patient temperatures and their neurologic status before and after the onset of the fevers and the outcomes after treatment.

Thirdly, we used fever as a presenting symptom for a possible UTI in this study. This was done as most neurosurgical patients have altered mentation and are not able to express alternative symptoms. Additionally, the retrospective nature of this study is limited by the thoroughness of the medical records, which may not include small subjective complaints, but commonly include objective findings such as fevers. However, this practice inherently excludes the individuals who would be able to express signs such a suprapubic tenderness and provide a higher clinical index of suspicion for urinary etiology of their fevers.

Fourthly, surgical patients are commonly placed on peri-operative antibiotics, usually either cefazolin or vancomycin, which could potentially interfere with certain culture speciation. We do not believe this to be a large limitation, as this would likely prevent from a fever occurring for a UTI if the organism were being adequately treated.

Lastly, our proposal to increase the threshold of obtaining urine studies in the setting of a new-onset fever comes from a small sample size. An analysis of a significantly larger population of neurosurgical patients with CAUTIs would better inform the validity of our proposal.

## Conclusions

Analysis of our patient population shows evidence to support the newest IDSA guidelines that patients who develop elevated temperatures should have a clinical workup of all alternative etiologies before testing for a urinary source unless the clinical suspicion is high. Further research is needed to evaluate for possible predictive characteristics of neurosurgical patients that have true CAUTIs. This will help reduce the rate of unnecessary urine cultures, the over-diagnosis of asymptomatic bacteriuria, the overuse of antibiotics leading to the development of MDROs and C. diff colitis. Based on our current findings, all potential sources of fever should be ruled out before obtaining urinalysis, and catheters should be removed as soon as they are not needed. Urinalysis with reflex to urine culture should be reserved for those cases where there remains a high index of clinical suspicion for a urinary source.
